# Stories and metaphors in the sensemaking of multiple primary health care organizational identities

**DOI:** 10.1186/1471-2296-15-41

**Published:** 2014-03-04

**Authors:** Charo Rodríguez, Emmanuelle Bélanger

**Affiliations:** 1 Department of Family Medicine, Faculty of Medicine, McGill University, 5858 Côte-des-Neiges Boulevard, Montreal, Quebec H3S 1Z1, Canada

## Abstract

**Background:**

The Quebec primary health care delivery system has experienced numerous reforms over the last 15 years. In this study, we sought to examine how managers and primary care providers made sense of the creation of successive new primary care organizational forms.

**Methods:**

We conducted a longitudinal qualitative case study in a primary care practice group located in Montreal, Quebec, for over 6 years (2002 to 2008). The data sources for the study include 31 semi-structured interviews with key informants, in-situ observations of group meetings, as well as documents and field notes. Textual material was submitted to narrative and metaphor analysis.

**Results:**

The core metaphor of the *journey* came from a set of stories in which the members of this primary care group depicted the processes undertaken towards developing a multidisciplinary cooperative practice, which include an uneasy *departure*, uncertainty about the *destination*, conflict among members who *jump ship or stay on board*, negotiations about the *itinerary*, and, finally, enduring challenges in leading the way and being *pioneers* of change in the organization of primary care in their institutional context. Identification with the initial family medicine unit identity was persistent over time, but successive reforms further enriched its meaning as it became a multidisciplinary primary care practice pioneering organizational change.

**Conclusions:**

In order to support primary care reforms in complex institutional fields, this study proposes that decision-makers undertake a journey in which they recognize both the need to capitalize on existing meaningful and legitimated organizational identities, as well as the necessity for collective leadership in the management of multiple organizational identities over time.

## Background

This paper examines how primary care managers and practitioners create and recreate their organizational identity in complex and challenging situations of change. More particularly, the study aims to better understand how organizational members operating in a public health care context experience the creation of successive new primary care organizational forms.

As in many other Western countries, health care delivery systems across Canada have been subjected to continuous reforms over the past few decades. A common characteristic of these recent systemic changes across Quebec and other Canadian provinces has been the emphasis on front-line services [1-4].

Under the publicly funded Quebec Medicare program, and before policy shift towards multidisciplinary team-based practices, primary medical care was generally provided by solo practitioners working in clinics or polyclinics and remunerated on a fee-for-service basis. A minority of physicians worked in community-care centres (*centres locaux de services communautaires* or “CLSCs”) or in family medicine units (training facilities for family medicine residents), the latter being located both in hospital and CLSC settings. In order to overcome pervasive problems of accessibility and continuity of care, the Quebec government initiated a reform process in 2002 involving the creation of new organizational forms called “Family Medicine Groups” (henceforth “FMGs”) [5]. FMGs include physicians, nurses and administrative staff working together for a rostered population to whom they provide a range of front-line preventive and curative services. A few years later, the limited reform’s success in the Montreal metropolitan area [6] encouraged the Health Regional Board to develop the concept of “Network Clinics” [7]. These facilities are walk-in clinics that offer medical services at a minimum of 12 hours per workday and 8 hours/ per day on weekends, 365 days a year. In addition, physicians have rapid access to laboratory and imaging services, and in certain cases, other technologies such as ECG, spirometer, and so forth. This new organizational structure was meant to facilitate access to care for the whole population of a given territory (i.e. not only to rostered patients), as well as to improve coordination across secondary and tertiary care.

Nowadays, as a result of these successive reforms, the same primary care practice can be a Family Medicine Group *and/or* a Network Clinic. These new administrative organizational forms overlap with former medical clinics, family medicine units and community care centres. While policy-makers, in the exercise of their authoritative power, may instigate change defining structural features of new organizational forms and set health care systems goals to be reached, organizational members (i.e. managers and providers) have to make sense of those changes in their day-to-day work-life in order to put policy reforms into motion. In other words, policy-makers may offer a general framework for the reform (in this case, the creation of new team-based primary care organizations), but those who will effectively bring those new organizations into being are primary care managers and providers. These reforms therefore concern *the emergence of new organizational identities*. In such a complex and “pluralistic” institutional context shaped by the divergent goals and interest of different internal and external organizational stakeholders [8], the research question guiding the present study was the following: *How have primary care providers and managers constructed their multiple organizational identities over time?* This represents an important issue to explore because identity and organizational members’ identification with organizations are processes associated with a large range of organizational outcomes [9,10]. What is more, the construction of the organizational identity of multidisciplinary primary care practices is an underexplored topic in today’s health services research literature [11].

### Conceptual framework

Identity is one of the major topics of examination in sociology. While the focus was initially on individual identity, collective identity has attracted special interest over the past few decades [12,13]. *Organizational identity* is considered a form of collective identity [14-16]. In organizational and management literature, organizational identity is viewed either as a central and enduring feature of the organization [17] or as a singular organizational process under constant re-creation [18]. Despite ontological differences, scholars consider that organizations may display multiple identities [19] that “can and should be managed” [20]. Indeed, it is also agreed that the organizational sense of self (or selves) is disrupted during periods of change: for organizational identity to come into being, organizational members have to make sense of it, and “[f]or organizational change to take effect, it should be appropriated by members and therefore must be accompanied by change in members’ identifications” [21].

**Figure 1:**
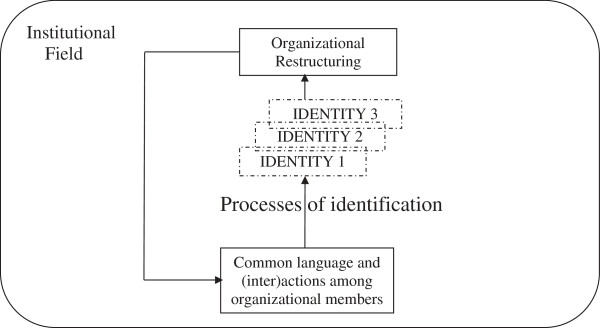
Making sense of multiple organizational identities in a public health care field.

Identification processes refer to communicative activity by which organizational members adhere to or distance themselves from emergent identities [21-23]. Accordingly, organizational identity can be disclosed through narrative structuring processes, i.e. identification processes emerging from interactions among organizational members [24,25]. Furthermore, these organizational processes occur within an institutional field in which change can be triggered in different ways through “identity threats”, i.e. potential disruptive events that bring organizational members to question their sense of self as an organization [26,27]. In the Quebec public health care sector, where the present case study was conducted, the successive primary care reforms developed over recent years therefore constitute “identity threats” (see also Figure 1).

When considering identity construction (i.e. identification) as a narrative process, the role of discursive practices such as metaphor in the construction of new identities (i.e. organizational change) becomes prominent. Metaphors are linguistic devices that “convene new meanings by fitting them into imagination-stimulating meaning”, and do so by breaking “through old labels, creating a hope for change, for something new” [24]. What is more, “a powerful metaphor initiates and guides social processes” [24]. Accordingly, metaphors will be involved in processes of identification resulting in new organizational identities [24].

## Methods

This paper draws on an in-depth longitudinal qualitative case study [28], which was conducted from the spring of 2002 to the fall of 2008. A longitudinal design was necessary to adequately gauge organizational change [29]. Approval from the McGill Faculty of Medicine Ethics Committee was obtained and annually renewed during the whole research period in question (A04-E08-03A). We also obtained timely approval from the ethical review board of the organization concerned.

The selected case is a primary care practice located in Montreal and was among the 3 initial pilot FMGs located in the region, and the one that had the most complex initial structure in comparison to more standard groups. In effect, at the beginning of the study, it included 21 family physicians (equivalent to 10 full-time) from both a *medical clinic* and a *university family medicine unit* (FMU) in a community hospital setting, 2 nurses considered as staff of the neighbourhood *community health centre*, and 2 administrative staff members. The group was also accredited as a *Network Clinic* in January, 2008. By the fall of that year, the organization had significantly increased its medical staff to 41 physicians.

The main method adopted for generating data was face-to-face, one-on-one interviews [30] with managers, physicians, nurses and administrative staff, conducted by both authors from the fall of 2002 to the summer of 2008 at different points in time: the first author was in the field during the first period of the study, while the second author completed the fieldwork during the last two years. Written informed consent for participation in the study was obtained from all the participants before conducting interviews (Additional file 1). Some key informants (i.e. group leaders, nurses) were interviewed 2–4 times as changes occurred in the group, and interviews were carried out with new members as personnel turnover occurred within the organization. In total, we conducted 31 interviews, lasting on average 1 hour: 6 interviews with 5 project managers at local and regional administrative levels, 7 interviews with the 2 physicians consecutively leading the group, 7 interviews with different FMG physicians, 6 interviews with the 3 nurses who practiced in the group throughout its history, as well as 5 interviews with 4 members of administrative or support staff. With the permission of the participants, interviews were recorded, transcribed and analyzed with the help of NVivo 7.0 research software.

On-site participant observations and field notes also constituted a valuable empirical technique [31] and complemented interviews. They refer mainly to a total of 22 inter- and intra-organizational group meetings, as well as observations in waiting rooms. Finally, documents and archival materials [32] (i.e., proceedings and minutes, formal agreements, media articles, government reports, etc.) were also sources of empirical material over the period of inquiry.

For the analysis of transcripts from individual interviews with key informants, we were particularly concerned with the interplay between stories and metaphors [33]. As noted by Gabriel [34]: “Narratives and stories feature prominently as sensemaking devices, through which events are not merely infused with meaning, but constructed and contested” (p. 62). Metaphors, in turn, are figures of speech that although not always used consciously, constitute roots of human knowledge [35], and as noted above, play an important role in processes of identity construction [36]. We thus proceeded as follows: throughout the entire research period, we separately read and re-read the whole corpus of empirical material gathered, i.e. conducted naïve reading [37]. This familiarization with verbatim transcripts, combined with our in-situ observations, field notes, and the documents gathered allowed us to understand the stories told by practitioners and managers of this primary care team about how they have dealt with the successive reforms. From this initial analytical step, we then performed a more structured analysis [37] by coding verbatim transcripts in order to inductively identify and develop the metaphors that fleshed out the meaning that organizational members gave to each process of identification. This analytical phase was performed by both authors separately on the material they had respectively gathered. A final story that included a core metaphor and significant sub-metaphors [38] which depict the whole process of organizational change finally emerged and was developed through successive discussions between us.

## Results

### The core metaphor: a journey through organizational change

The core metaphor of the *journey* emerged from the close interpretation of all the stories. This metaphor was not referred to explicitly by respondents, but their narratives clearly pointed to an *uneasy departure*, *uncertainty about the destination*, conflict among members who would either *jump ship or stay on board*, needs with respect to *redrawing the itinerary*, and finally enduring challenges in leading the way and *being pioneers of organizational change* in primary care. When asked to describe the evolution of the FMG, the group leader provided a long narrative, which is partly reproduced in *The core metaphor: a journey through organizational change* herein. Originally, primary care providers did not know what the role of different members involved in this new project would be, and there was considerable resistance to the project. However, toward the end of the study period, and despite lingering uncertainty about the possibility of further government reforms, a new FMG entity had merged with the organizational structure as a result of extensive negotiation and adaptation. The narratives supporting these stories were explored in depth and we discuss their relation with identification processes below.

#### *The core metaphor: a journey through organizational change*

“So it was 2003, the first leader negotiated with the regional agency of health services so that the FMG be created, March 2003. It was in October 2003 that our nurses, two nurses, started to work. Historically, they were… it was the very beginning of FMGs; they had to decide which patients they would see, the reference sheet they would use. Very few patients were referred to them, because the doctors had never worked obviously with references to nurses. […] It was clear that we needed to hire and keep people that we could trust. We changed one of our nurses at the beginning. […] We had nurses that were not very busy, and as physicians saw that they could trust them, and that they were doing good work, and that their patients were satisfied, then the rates of references increased, OK? It goes without saying that the evolution was also about convincing the reception that the FMG should exist. Because at the beginning there was an enormous amount of resistance, with the receptionists not agreeing with it, judging that it was merely an additional workload. […] So it was difficult to make receptionists and physicians realize the value of the FMG. And the physicians got on board, and recently the receptionists too, since we’ve become a Network Clinic. […] So what matters for patients is that we were able to recruit physicians, because they are interested in joining an FMG and that we can now increase the number of registrations” (FMG leader).

### Where to? Uneasy departure and uncertainty about the destination

Uncertainty was pervasive in the implementation of the FMG structure. Several aspects of the project remained obscure, such as who would be part of the group as it overlapped with different pre-existing structures, what the nurses’ role would be, how the new entity would be introduced to patients, and more importantly what would happen if the project was ever discontinued as part of never-ending government reforms. This uncertainty prevented the actions necessary to bring the FMG into existence, and it highlighted the initial absence of a coherent narrative that would establish a common destination and support the identity of the new organizational entity.

From the onset of the project at the study site, the complexity of the organization was obvious to everyone involved. To reiterate, the case study targeted a FMG of great complexity, integrating two medical sites with different traditions of practice, and whose members overlapped, albeit not completely. Not only was this FMG among the first to implement a multidisciplinary primary care practice, it was also facing the integration of geographically separate sites with different styles of practice (see excerpt 1 in *Uneasy departure and uncertainty about destination*). Recruiting physicians at the FMU and at the clinic was not an easy task for the group leader. Physicians remained wary of the thick contracts that had to be signed with government agencies, and wondered if all the advantages that they were contemplating would eventually cost them (see excerpt 2 in *Uneasy departure and uncertainty about destination*). At the beginning of the project, the FMG organization concept was not well-known and some people assumed that it would eventually disappear:

The physicians said to us: ‘You take new patients, and then the government decides that we stop this project while I’ve taken a lot of new patients. What will I do with all these patients then?’ So in fact it was as if we were all on hold. (FMG nurse)

The first major impact of the FMG was the arrival of the two nurses. Integrating them into the group and finding out what contribution they could make was not easy. “It was difficult at first, because we did not know what tasks we would give them and what they would be willing to do” (FMG physician). Then the group had to figure out how this would be explained to patients, and how it would affect their access to medical care depending on their being registered patients or not:

I don’t know if you read that pamphlet produced by the Ministry? It was quite incredible [*laughing*]. We changed it. You know, 24 hours a day, 7 days a week you will be able to see or talk to your physician, woh, woh, woh! The physicians got scared reading this; let’s not give this to anyone! (FMG administrative staff)

From its inception, the very purpose and development of the FMG was a moving target. The first few physicians who decided to implement the project, one of them being the first FMG leader, envisioned the FMG reform as a real opportunity to implement the ideal of cooperative practice:

In the past before, when I started practicing, we were four, five doctors on the 2^nd^ floor and we worked as a little polyclinic. We shared everything, we arranged duty hours, and we offered medical services from Monday morning to Friday night […] to everybody […]. Then, the offer of services became based on a solo practice. And each of us was convinced that we offered the best possible medical service. But it was an image in the water. Then for me it [*the FMG reform*] was a means for reintroducing a shared medical practice, a way to offer a better service to our patients, for establishing a more ideal model of practice. (FMG leader)

Yet they had to convince others to join in (“I went to see everybody, and despite my power of persuasion (…), people said to me: ‘No, [*name*], we respect what you do but we are not interested’” (FMG leader)), and to develop ways of working with clinical nurses. Every aspect of the group’s activities had to be determined, negotiated. At the beginning of the project, a shared goal was missing from members’ narratives, preventing any identification with the emerging structure (see excerpt 3 in *Uneasy departure and uncertainty about destination*). The adaptation was lengthy as with any organizational change, and some of the physicians expressed their dissatisfaction at the beginning of the project.

#### *Uneasy departure and uncertainty about destination*

##### 

**EXCERPT 1:** “That it is a FMU [teaching family medicine unit in a hospital] is really particular because it is enormously confusing, what falls under the hat of the FMG, and now they are also a Network Clinic. What is under the hat of the FMU, and what is under that hat of the networking clinic? So the physician in charge is freaking out a little, because he does not know how to manage his things. The physicians of the FMU who did not sign still benefit from the resources of the FMG; we cannot say do not ask my nurse because she is FMG. So they are sharing. So when we create new FMGs, well I strongly discourage clinics from becoming an FMG if it is not all of the physicians who are signing because it becomes chaotic; they have a deficit and have difficulty achieving registration targets. The administrative technician and secretaries are overworked and do not have time to manage registrations because their time was estimated on the basis of the physicians in the group, and in the end the real number of physicians is much larger than those who signed” (FMG regional manager).

##### 

**EXCERPT 2:** “I told the people: ‘I know, we know that there are too many unknowns and that it is stressful for you to register. You do not know what the consequences will be, although the registration does not commit you to much and we can always leave at any time. We will not force you to register. It will be completely voluntary and if you want, you can leave’. Obviously, nobody ran after me to say they wanted to register” (FMG leader).

##### 

**EXCERPT 3:** “Who are you? Where do you work? What’s an FMG? There is still some skepticism surrounding what the FMG is and what it will bring. We do not hear it as much, but people still say it sometimes: ‘That won’t work’. There is still a lot of misunderstanding about what it is and its use. From that point of view there is still a lot of work to be done” (FMG nurse).

### Jumping ship or staying on board: conflicting identifications

The complexity of the organizational structure undoubtedly had a lot to do with the problems that were encountered when trying to integrate a FMU and a clinic for certain administrative purposes while promoting multidisciplinary work. The former had a well-integrated educational mandate, yet adding the FMG structure on top of it was perceived as an additional burden by much of the support staff, who proved uncooperative until they were included in the project and until the contribution that the nurses could make in terms of continuity of care was explained to them (see excerpt 1 in *Jumping ship or staying on board: conflicting identifications*).

The conflict with the support staff of the FMU was perhaps the most significant barrier to functioning as an FMG. Patients had to be registered, which represented more administrative work for an overburdened team who could not perceive the benefits:

The secretaries there [FMU] were unionized, so the added FMG tasks were perceived as something external; it was hell for many years. I had to print myself and fill-out forms myself because the secretaries at the FMU did not want to do it. It was a complete boycott. (FMG physician)

#### *Jumping ship or staying on board: conflicting identifications*

##### 

**EXCERPT 1:** “I think that they had difficulties exactly because of the teaching mandate, which is a mandate that works well and that is well integrated into the work of all employees including the support staff. It has been existing for a long time, and it works well. So I think that when the FMG was added to the team, it was difficult for them. It started with difficulty. It was hard to integrate and harmonize both mandates: the FMG mandate and the teaching mandate” (FMG regional manager).

##### 

**EXCERPT 2:** “What I needed in primary care was someone to help with flu shots for example, something really simple like that. I did not have anyone. I had a nurse following my chronic cases, and I had to do the flu shots. It’s been like that for a long time and it makes no sense. I yelled against that but it was no use talking against the nurses, they were clinicians. But that’s not what I needed. I needed an auxiliary nurse. That’s what I needed. And I needed a secretary to register my patients; I shouldn’t be the one filling that out” (FMG physician).

Whether or not FMG patients should be prioritized in walk-in clinics was a continued topic of debate in FMG administrative meetings, and it was yet another source of conflict with the support staff. They were the ones faced with refusing patients based on their FMG status, and they did not feel comfortable doing so:

I don’t agree about the walk-in clinics. Prioritize the patients for some appointments, for follow-ups. Because you are not FMG, you won’t get to see the nurse. That I can understand; it is hard to live with, but I can still tell them. But don’t go refusing patients at the walk-in clinic because they are not FMG. In any ways, they will not have my collaboration for that. (Support staff)

As the excerpt below illustrates, some physicians even decided to withdraw from the FMG when they realized the bureaucratic weight that it represented and the prolonged adaptation that was required to really integrate the nurses in such a diverse context of practice:

At the beginning I was for it, I joined in, and we had one nurse and then that first nurse that we had hired took about 6 months to look at the resources. She had not even seen a single patient I think; it was ridiculous. […] So I decided to quit, because it made no sense, as a protest. (FMG physician)

After the renewal of the first three-year contract, a new physician took over the leadership of the group and decided not to renew the contract of one of the nurses who had been experiencing difficulty in her interactions with physicians. She was replaced with someone perhaps more inclined to adapt to the various styles of practice of the physicians:

She was much more confrontational with regard to her professional position. Physicians do not want to work with nurses; that was her way of seeing things. She told me from the start, “I think that physicians join the FMG because it’s lucrative”. With that attitude, of course it very quickly became confrontational, so I can understand that after some time it wasn’t working at all. (FMG physician)

There were also disagreements in terms of what the role of the nurses should be. The ones who were designated to join the project were experienced and had extensive clinical training. They could thus follow their own clientele of chronically ill patients after a referral by a physician. However, some physicians, while recognizing that nurses were a valuable addition to the services offered on the sites, believed that the most pressing tasks for less qualified personnel were to take simple measures and administer injections so as to free them up to take on more complex tasks (see excerpt 2 in *Jumping ship or staying on board: conflicting identifications*). Other physicians, on the other hand, would have preferred an even more independent professional, one with a role closer to that of a nurse practitioner. They argued that a professional with that role could have solved many of the simple problems that presented themselves at the walk-in clinics.

In terms of identification processes, having members abandon the group contributed to the polarization of allegiances and to the exposition of conflicting visions in terms of the destination of the FMG. Until all the members reached a consensus on the value and meaning of the FMG, it was not possible to develop a strong identity in a group whose continued existence appeared so fragile.

### Redrawing the itinerary: negotiations

The members of the FMG went a long way in negotiating the role of the nurses that joined the group and in developing more spontaneous patterns of cooperation. Eventually, the clerical support staff also came to terms with the bureaucratic weight of the registration process. This was not achieved overnight, however, and great efforts in negotiation and dialogue were necessary to steer the group in a common direction. For instance, the group leader emphasized the need to remind physicians of the nurses’ role in order to increase the number of referrals (see excerpt 1 in *Redrawing the itinerary*). The number of referrals increased after the nurses threatened to leave:

We met them and said, if things keep going the way they are, by fall there will probably not be any nurses in your FMG. For the first time they really reacted: ”why?” One said: “I’m sorry, I am in the FMG yet I have never referred you any patients. I’m used to sending them to the day clinic”. Another one said: “That’s true; I’ve been acting as if I wasn’t FMG, as if it didn’t exist”. Yet another said: “I do not really know what you do. Other than just filling out orders, are you able to do more?” And then they said they didn’t want us to leave, that they had nothing against us. From then on, we started moving forward. (FMG nurse)

#### *Redrawing the itinerary*

##### 

**EXCERPT 1:** “I told the nurses: the first ten minutes [*of the FMG meeting*] you will present what you do. You will put that on a PowerPoint. It is of interest for the physicians. Ten minutes, no politics, nothing on the law, only what you do, what patients you are seeing. Because they will realize what you are able to do. Then they will click and refer patients to you. But if you do not sell your skills, they do not know what you do and they will not send patients. So at every meeting you will come, so that the physicians get used to sending you patients” (FMG leader).

##### 

**EXCERPT 2:** “I think that physicians noticed the advantages of being part of a family medicine group. The government encourages us to use administrative hours to enable us to count the hours spent calling our patients, or when we work on computers to improve our patients’ prescriptions. That is work that wasn’t paid when we worked in private offices, while now we have a number of administrative hours, which obviously makes practice more lucrative. Those are things that slowly, as we informed the physicians of these FMG advantages, they started realizing that it was worth it. Other advantages were the FMG computers that were provided, which enabled some of them to have a computer-based practice” (FMG leader).

With time, the advantages of being an FMG were recognized by everyone in the emerging organization. The support staff became better at convincing patients of its value, so the rate of registration improved dramatically. Nearly all the physicians teaching residents joined in order to facilitate the administration of the service provisions (see excerpt 1 in *Redrawing the itinerary*).

Towards the end of the field research, many physicians admitted that having a nurse in the practice was a clear advantage for their patients with chronic conditions. With respect to the level of cooperation between nurses and physicians, most members would agree that the implementation was slow, but ultimately successful:

Like I said, initially, it was not obvious. They [nurses] were welcomed with a little suspicion. […] What happened is that as we saw the excellent quality of care provided by the nurses, physicians became reassured. And it became easier to use the help of nurses. So when we talk about integration, really now we can say that they are part of the team. (FMG leader)

Indeed, most of the physicians who had “jumped ship” decided to reintegrate the group once roles were formalized and cooperation became more fluid. They started to build a common ground for an emerging organizational identity, which nonetheless developed only slowly. The FMG remained only one of the overlapping groups with which members could identify.

### Being pioneers of organizational change: the enduring challenges of multidisciplinary practice

It appears from the data that FMG members eventually came to recognize both the value and limitations of the new organizational form. Identification therewith was mitigated by a strong pre-existing identification with the FMU teaching mandate, by new bureaucratic burdens, and by the limited resources that came with the entity, which remained mainly administrative in purpose: “Of course that was the idea, to see more patients as our nurses get here, but one nurse? Two nurses for the equivalent of 10 full-time physicians? It is not much” (FMG leader).

Upon realizing that the FMG was not a group that was readily mentioned by members when describing their practice and allegiances, the group leader expressed no surprise. The two separate geographic locations also accounted for the group’s abstract existence: “The FMG is virtual because the FMG has not really changed the services in general that the population has been receiving. It did not change the way physicians practice” (FMG leader).

The real success of the project in terms of promoting member identification was the productive cooperation that eventually developed between physicians and nurses. Despite initial difficulties, this was a matter of pride for members of the group who realized how it could improve continuity of care for chronically ill patients: “The biggest success of the FMGs is to have physicians and nurses working together. That worked; it works well, not everywhere but it was a big challenge. Indeed, that is the success” (Project manager). Although the physicians were pleased with their achievements in terms of cooperating with the nurses, they did not exhibit a great attachment to the administrative structure that had supported the change:

The FMG, besides the nurse, did not bring that much more to our FMU. It was already team work. It forced us to have someone on call on weekends, which we didn’t have before, so it changed some ways of working. […] It did not bring an enormous change, but I think that it did consolidate multidisciplinary teamwork and the cohesion of the team. (FMG physician)

It is important to mention that, in this primary care practice, physicians had always been involved in teaching and been supportive of innovative modes of medical practice. Hence, despite initial difficulties, there was a great deal of support for greater cooperation between nurses and physicians. The essence and vision of multidisciplinary cooperative practice at the primary care level rallied nearly all respondents, while its administrative form of overlapping structures did not. Indeed, being among the first FMGs did create a sense of pride in its members, who identified themselves as being instigators of new modes of organization in primary care delivery. “I can see that it becomes less and less obscure at the level of the FMG itself. The fact that we were one of the first ones makes us pioneers. We are a resource for other groups” (FMG administrative staff).

The last meeting that we attended as part of field observations regrouped all the different practitioners that offer services at that location, and it was labeled the “FMU/FMG/NC meeting”. The FMG project thus successfully directed primary care toward more multidisciplinary work and toward more integration in the health network. The practitioners nonetheless remained highly attached to prior organizations, namely the FMU with its teaching mandate, which was responsible in the first place for attracting innovative clinicians who greatly value renewal of organizational structures aimed at improving primary care delivery.

## Discussion

The present study aimed to understand how, in a public health care institutional field, primary care organizational members made sense of successive waves of health care reforms. Our focus was on processes of organizational identification, considered as narrative processes, and on the role played by metaphors as powerful discursive strategies able to support change, i.e. the restructuration of collective interpretive schema (or identities). In this case, “identity threats” came from the institutional field, where public decision-makers had proposed successive models of primary health care organization that could be incorporated in professional identification.

Over the six-year period considered, these professionals made sense of the successive organizational transformations that they experienced by remaining attached to their original organizational identity, all the while undertaking a process of incremental change. The main metaphor that captures this process was *the journey*. The bulk of the members of the new FMG organization were already practicing in the FMU, an organizational identity they were strongly identified with. In the early days of the reform, this robust identification with the “initial” organizational form made the journey towards the appropriation of the new FMG interpretive schema very difficult. As noted by Chreim [21], high levels of members’ identification with prevailing identities may imply additional difficulties to implement successful change. These difficulties were initially overcome by a visionary group leader. Following his guidance, organizational members finally recognized the new model and they decided to initiate the journey by “formally” adhering to it, i.e. by signing the contract with the government. However, they continued to identify first and foremost with their original organizational identity: it was an uneasy departure with an uncertain destination.

The key element of the new organizational form was the integration of nurses throughout the evolution towards a multidisciplinary group of practice. Inter-professional conflict immediately arose, with members of the group having to decide between jumping ship or staying on board. The second FMG leader, appointed in 2005, successfully emphasized the added value of the new organizational form by providing a sense of continuity during the change, thereby accomplishing what Chreim [21] has labelled “confluence”. In other words, while the new leader emphasized the importance of the original organizational identity and insisted on the fact that a multidisciplinary primary care practice would do nothing but improve organizational performance, she was also able to create spaces of exchange and communication between physicians and nurses (discussions about journey itinerary) that made the emergence of an “additional” new group identity possible [39].

Although strong identification with the FMU prevailed, the process of change undertaken not only “enriched” the original organizational identity with new members (i.e. nurses) and additional material resources, but also fostered a self-image as pioneers in establishing new primary care organizational forms among its members. This resonated with their institutional context, thus giving legitimacy to the new FMG identity. This was further increased when, due to the university-affiliated character of their FMU, they were selected by policy-makers as a new Network Clinic.

This study first empirically demonstrates how persistent an organizational identity can be when it is highly legitimated in its institutional domain. When organizational members identify strongly with an organizational form, they are resistant vis-à-vis successive waves (organizational threats) of reform. That being said, our study also suggests that, while enduring, this initial organizational identity is also malleable over time. Indeed, capitalizing on strong pre-existing organizational identities may also facilitate adaptation to change and promote members’ identification processes with new organizational identities (multidisciplinary teamwork) that, in fact, can even increase their organizational legitimacy (pioneers in the implementation of new organizational forms). Drawing on Weick’s and Whitehead’s works, it is what Bakken and Hernes refer to when they stress that “organizing is both a noun and a verb” [40], that is to say that organizations are neither just entities (nouns) nor processes (verbs) but both entities and processes recursively related. The result is organizational identity congruence [41].

Second, this work demonstrates the crucial complementary role played by leaders with different managerial styles in the journey to create and manage multiple primary care organizational identities. In order to instigate organizational change, the effective leader of a group of professionals strongly identified with the existing organizational identity has to be a visionary who “sees” what the group cannot, or does not want to envision; someone who strongly believes in the value of the destination and even fights with the rest of organizational members to initiate such an uncertain journey. Then, once the process has been initiated, another leader with a more down-to-earth approach appears necessary to deal with the day-to-day obstacles encountered during the trip, someone who is able to keep the ship afloat. Both profiles are necessary for success, but at different moments in time. It is what has been called collective leadership [42]. As Baker and Denis [43] note: “While individual capabilities and qualities of aspiring leaders certainly contribute to organizational achievements, effective leadership in this view needs to be seen as an organizational or system property. Accordingly, the emphasis should be put on the development of groups of leaders within organizations that combine the diversity of expertise, skills and sources of legitimacy to respond to system challenges” (p. 358).

Our study makes a number of contributions for both research and practice. It first significantly enriches the health services research literature, dominated by functional approaches, with an empirical longitudinal qualitative study on organizational identity. In this study, the combination of metaphor and narrative analysis has helped us shed light on the very complex processes of healthcare reform —a methodological approach rarely employed even in organizational and management literature [33].

It will also help future policy and management decision-making with regard to the creation of new primary health care organizational forms. Although the evaluation of the implementation and effects of the first Quebec FMGs seems encouraging [6,44,45], decision makers should take into account that professionals have to make sense of new organizational forms in order to meet policy expectations. Our investigation proposes emphasizing existing meaningful and legitimated organizational identities, and then managing multiple organizational identities through effective collective leadership.

As for any investigation, concerns about the credibility (internal validity) and transferability (external validity) of this study may arise. First, in order to strengthen its credibility, we: (a) put emphasis on the methodological coherence between the research questions and the selected methods; (b) triangulated methods for generating data; (c) adopted concurrent processes of data collection and analysis; (d) put particular care to include not only sufficient but a considerable variety of key informants in the study; (e) adopted member checking to validate whether participants’ perspectives were adequately understood and interpreted. The transferability of the results from this single case study is in turn supported by: (a) the in-depth description of our case which helps the reader to better appreciate the influence of the case’s context; and (b) the critical nature of the case in question, a particularly complex FMG whose structure and organizational dynamics were very different from more typical FMGs.

## Conclusions

In the present investigation, we carried out a longitudinal assessment of the processes whereby members of a primary care practice made sense of multiple organizational identities emerging from successive waves of health care reform. To do so, we put methodological emphasis on understanding how stories and metaphors helped to construct organizational identities over time. Carried out in a complex institutional context, our study reveals the value of capitalizing on the most legitimated organizational identity while undertaking organizational change incrementally. As our study also demonstrates, this process of constant construction and reconstruction of meaningful organizational identities requires collective leadership; that is, different profiles of organizational leaders at different moments in time to successfully sustain organizational members through the journey in making sense of their multiple organizational identities.

## Competing interests

The authors declare that they have no competing interests.

## Authors' contributions

The first author developed the concept for the study and carried out the fieldwork over the first years of the research period considered. The second author was in the field over the last two years of inquiry. Both authors worked together in the analysis of data gathered, drafted the successive versions of the paper and approved the final version text.

## Pre-publication history

The pre-publication history for this paper can be accessed here:

http://www.biomedcentral.com/1471-2296/15/41/prepub

## Supplementary Material

Additional file 1 Interview guideline for family physicians and nurses of the FMGs. Click here for file
