# Supplementary material for: Stories and metaphors in the sensemaking of multiple primary health care organizational identities
Source: BMC Fam Pract. 2014 Mar 4;15:41. doi: 10.1186/1471-2296-15-41 (PMC4015182; doi:10.1186/1471-2296-15-41)
Supplement: Additional file 1 — Interview guideline for family physicians and nurses of the FMGs. [file 1471-2296-15-41-S1.doc]

**Interview Guide for FMG Physicians and Nurses**

**Brief introduction of the research project and consent forms**

**Professional Experience and FMG Membership**

1. Could you describe your professional background and experience?

2. Are you a member of the Family Medicine Group? When did you join the group?

3. Are you practicing full-time at the FMG? How many patients (and vulnerable patients) are registered in your name? Are you also affiliated with the Family Medicine Unit, the private clinic, the Community Health Center (CLSC)?

4. Could you describe your practice outside of the FMG? Are you in contact with other FMGs in your practice?

5. What motivated you to become a member of the FMG?

6. How has your practice changed since you became a member of the FMG? Do you now work with a nurse/with other physicians?

**Identity and Overview of the Activities of the FMG**

7. Knowing that the creation of the FMG was the only recommendation of the Clair report (December 2000) to be adopted and that the vision of the FMGs was very idealist at the time of their creation, eight years later, in your opinion what is a FMG now?

8. Could you describe your FMG from your own perspective; what does it represent?

9. Could you describe the distinctive characteristics of your FMG? How is it different from others in the context of primary care delivery in Montreal?

10. Could you summarize the evolution of practice in the FMG since the first three-year contract, up until the current contract that began in March 2006?

11. To this day, what are the resources that belong specifically to your FMG? Could you describe some of the decisions that the FMG has made?

12. What are the activities that have been developed by the FMG? Could you specify the activities and contacts linking your FMG and the private clinic, the Family Medicine Unity, and the Community Health Center?

13. Physicians: What is your perception of the integration of nurses in your FMG?

Nurses: What is your perception of the collaboration between physicians and nurses in your FMG?

14. Do you feel a sense of belonging toward the FMG? Is it important for you to be part of this group?

**Image of the FMG**

15. In your opinion, what do other members think of the FMG? Have you noticed any differences between the physicians working at the clinic and those working at the Family Medicine Unit?

16. In your opinion, what do medical staff members who are not part of the FMG think of this group? In terms of attitudes toward the FMG, do you think that there are any differences between the staff at the private clinic, the Family Medicine Unity, and the Community Health Center?

17. In your opinion, what do people at the Community Health Center think of the FMG?

18. In your opinion, what do patients (registered and non-registered) think of the FMG?

**New Institutional Context**

19. Many changes have occurred recently in the institutional context of the FMG, for example, the creation of a new Center of Health and Social Services and of an Integrated Network Clinic at the Family Medicine Unit. According to you, what have been the consequences of these changes for the FMG?

20. Could you describe the activities and contracts linking the FMG and these new structures (Center of Health and Social Services, Network Clinic)?

21. What have been some of the successes and failures of the FMG in this new institutional contact?
